# Involvement and burden of informal caregivers of patients with mental illness: the mediating role of affiliated stigma

**DOI:** 10.1186/s12888-023-04553-x

**Published:** 2023-01-26

**Authors:** Mark Mohan Kaggwa, Sarah Maria Najjuka, Mohammed A. Mamun, Mark D. Griffiths, Novatus Nyemara, Scholastic Ashaba

**Affiliations:** 1 Department of Psychiatry, Mbarara University of Science & Technology, Mbarara, 1410 Uganda; 2 African Centre for Suicide Prevention and Research, Mbarara, 379 Uganda; 3 Department of Psychiatry and Behavioural Neurosciences, McMaster University, Hamilton, Canada; 4 Makerere University, College of Health Sciences, Kampala, Uganda; 5 CHINTA Research Bangladesh, Savar, Dhaka 1342 Bangladesh; 6 Department of Public Health and Informatics, Jahangirnagar University, Savar, Dhaka 1342 Bangladesh; 7 Psychology Department, Nottingham Trent University, 50 Shakespeare Street, Nottingham, NG1 4FQ United Kingdom

**Keywords:** Affiliated stigma, Informal caregivers, Mental illness, Marital status, Caregivers’ burden, Caregivers’ involvement

## Abstract

**Background:**

The fear and lack of understanding of mental illness can lead to stigma. The stigma of mental illness affects not only individuals who suffer from it, but also the caregivers. Stigma among caregivers can lead to delay in seeking care, poor adherence to treatment and a high risk of relapse. Caregivers of patients with mental illness are at an increased risk of distress due to the burden to stigma and caregiving burden. An increase in caregivers’ burden can lead to a reduction in caregivers’ involvement. There is a relationship between caregivers’ involvement, burden, and affiliated stigma. The present study examined the mediating role of affiliated stigma in the relationship between caregivers’ burden and involvement among informal caregivers of hospital-admitted patients with mental illness in Uganda.

**Methods:**

A cross-sectional study was conducted among 428 informal caregivers (mean age: 39.6 years [SD±14.6]; females = 62.1%). Information was collected regarding sociodemographic characteristics, affiliated stigma, and the involvement and burden of informal caregivers.

**Results:**

The findings indicate that affiliated stigma serves as a full mediator between the caregiver’s roles and involvement (β=15.97, *p*<0.001). Being female increased the caregivers’ burden of caregiving (β= -0.23, *p*<0.001).

**Conclusion:**

The findings in the present study suggest that intervention to address affiliated stigma among caregivers of patients with mental illness should be incorporated into mainstream mental health care to reduce the caregiving burden.

**Supplementary Information:**

The online version contains supplementary material available at 10.1186/s12888-023-04553-x.

## Introduction

Fear or lack of understanding of mental illness can lead to stigma [1, 2]. In mental health contexts, stigma refers to the act of discrediting, devaluing, and shaming a person because of mental health illness [2, 3]. The stigmatizing acts can also involve prejudicial attitudes, stereotypes, discriminatory behaviors, and biased social structures endorsed by others about mental health illnesses or individuals who have mental illnesses [4, 5]. There are three types of stigma, (i) public stigma: prejudice, stereotypes, and discriminatory attitudes towards people with mental health illnesses, (ii) self-stigma: internalized shame that people with mental health illnesses have about their own condition, and (iii) institutional stigma: where organizations intentionally or unintentionally limit opportunities for people with mental health illness) [3]. In addition, another type of stigma has been reported among the associates of people with mental health illnesses, such as caregivers [6]. This is a form of internalized stigma known as affiliated stigma, comprising three inter-connected psychological responses (i.e., affective response – such as anger and despair; cognitive response – such as feeling inferior to others; and behavioral responses – such as withdrawing from social relationships or feeling alienated [4]).

There is a positive association between affiliated stigma and the burden of caregiving has been documented in previous research [7]. In addition, there is an association between affiliated stigma and caregivers’ burden [8] while at the same time, a higher caregiving burden is related to more severe affiliated stigma [9]. Therefore, higher levels of affiliated stigma may result in caregivers perceiving a greater caregiving burden. Similarly, caregivers with many burdens can feel guilty because they think they are providing inadequate care to the individuals they are looking after. This process involves cognitive, emotional, and behavioral reactions that could lead to greater affiliate stigma levels.

Apart from the burden of caregiving, caregivers also experience other negative subjective appraisals that may lead to affiliated stigma. This includes daily care bother (arising from assisting with the activities of daily living such as assistance in patient hygiene [bathing, dental hygiene, nail and hair care, etc.], instrumental activities of daily living such as paying bills), and behavioral bother (arising from mental health illness patient behaviors such as violence towards caregivers, destruction of property, wandering, etc.) [10, 11].

When some caregivers are involved in the care of individuals with a mental illness, the most reported emotional reaction is a shame [8, 12–15]. The greater the shame, the higher the levels of affiliated stigma and burden of care [8, 15]. In addition, greater levels of affiliated stigma and caregivers’ burden are associated with lower exemplary care [10]. Exemplary care is a construct used in assessing the quality of patient care/caregivers’ involvement that relates to communicating to care recipients that they are loved, respected, and worthy of special consideration [10, 16]. At the same time, decreased caregiver involvement is a known consequence of affiliated stigma [17]. However, caregiver involvement plays an integral role in an individual’s recovery by supporting them financially, providing housing, offering emotional support, supervising them, and supporting them to take better care of themselves [18].

**Fig. 1:**
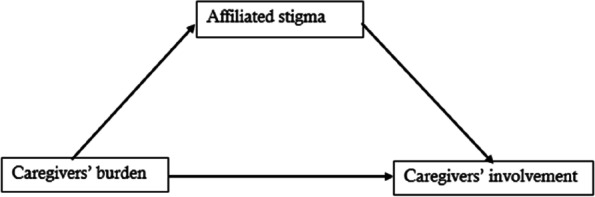
The proposed theoretical model

Based on the existing literature [19, 20], the burden of caregiving can lead to a reduction in caregiver involvement. Due to the association between affiliated stigma, caregiver involvement, and caregiver burden documented in the existing literature, it was hypothesized that affiliated stigma plays a mediating role in the relationship between the participation and burden of caregivers (Fig. 1 – model proposed by the present authors). To our knowledge, this relationship has not been previously investigated in Uganda. Therefore, the present study investigated the mediating role of affiliated stigma in caregiver burden and involvement relationships among caregivers of patients with mental illness in southwestern Uganda.

## Methods

### Study design and setting

This cross-sectional study was conducted among caregivers of patients with mental illness admitted at the Mbarara Regional Referral Hospital (MRRH) psychiatry unit between July and November 2020.

### Inclusion and exclusion criteria

Eligible participants were caregivers aged 18 years and above. Caregivers who were physically ill or unable to communicate verbally and comprehend the contents of the consent document due to intellectual disability or cognitive disability were excluded from participation.

### Sample size and sampling

The sample size was calculated using the Epi Info StatCalc for Population Surveys (Version 7.2.2.6). The calculation used a population size of approximately 800 caregivers, power of 80%, expected frequency of severe affiliated stigma at 50% (because no recent study concerning affiliated stigma has been carried out among caregivers in Uganda, and a value that maximizes sample size was used) [21], an acceptable margin of error of 5%, and a design effect of 1.0. Consequently, the calculated sample size was 385. With adjustments for non-respondents of approximately 10%, the sample size aimed for was 424 participants. The data were collected utilizing convenience sampling.

### Data collection

Research assistants approached potential participants on the day the patient was discharged from the hospital. Caregivers who showed willingness to participate in the study were given a consent form to sign before enrolment. Three trained research assistants with expertise in mental health data collection administered the pretested questionnaire to the participants to collect data through face to face interviews. During the data collection process, there was a 100% response rate among those approached.

### Study measures

#### Basic information of caregivers

The authors developed a questionnaire that included items on socio-demographic information: age, gender (male vs. female), address (rural vs. urban), marital status (single, married/cohabiting, divorced, separated, widowed), level of education, number of children, area of dwelling (urban vs. rural), birth order, monthly income (in USD), number of patients with mental health illness under their care, and duration of care in years. In addition, the diagnosis of patients of the caregivers was recorded from the patient’s hospital records and was classified in accordance with the ICD-11 classification of diseases.

#### Caregiver involvement

Caregiver involvement was assessed using the Involvement Evaluation Questionnaire (IEQ). The scale was developed in 1987 and finalized in 1992 [22]. It is a self-report 33-item scale, and the first 29 items aggregate into four distinct sub-scales while the last four items form a ‘generic’ sub-scale as observed in the previous four weeks. The sub-scales include tension (nine items; e.g., *“Has your relative/friend disturbed your sleep?”*), supervision (six items; e.g., *“Have you guarded your relative/friend from committing dangerous acts?”*), worrying (six items; e.g., *“Have you worried about your relative/friend’s safety?”*), and urging (eight items; e.g., *“Have you encouraged your relative/friend to undertake some kind of activity?”*). It has a high test-retest reliability of at least 0.7 and response rates to the IEQ have tended to be high (70%+), and the resultant data tend to be completed with minimal missing values [23]. Each question is answered on a 5-point Likert type scale from 0 (*never*) to 4 (*always*). The mean score for each sub-scale represents the severity of caregivers’ involvement. In the present study, the Cronbach alpha of the sub-scales were 0.87 (tension), 0.77 (supervision), 0.81 (worrying), and 0.86 (urging), and 0.92 for the total scale.

#### Caregiver burden

Caregiver burden was assessed using the Zerit Burden Instrument. The scale comprises 22 items that assess the level of care burden, with a total score of 88 [24, 25]. Each item on the scale is a question (e.g., *“Do you feel that because of the time you spend with your relative that you don’t have enough time for yourself?”*) that the caregiver is asked to answer using a 5-point Likert-type scale. Response options range from 0 (*never*) to 4 (*nearly always*). Higher total scores indicate higher levels of caregiver burden. Scores are classed into four categories; (i) little or no burden (0-20), (ii) mild to moderate burden (21-40), (iii) moderate to severe burden (41-60), and (iv) severe burden (61-88). The tool was previously used in a similar setting (Nigeria) [26]. The tool has excellent reliability (between 0.85 and 0.93) [23]. In the present study, the Cronbach alpha was 0.93.

#### Affiliated stigma

The Affiliate Stigma Scale was used to assess the self-stigma of caregivers providing care to family members with a mental health illness or intellectual disability [4]. The instrument has 22 items rated on a 5-point Likert scale across three domains; (i) cognition (seven items; e.g., *“My reputation is damaged because I have a family member with mental illness”*), (ii) affect (seven items; e.g., *“I feel emotionally disturbed because of my family member with mental illness”*), and (iii) behavior (eight items; e.g., *“I have cut down on going out with my family member with mental illness”*). The scale is scored from (*never*) to 4 (*nearly always*). Higher total scores indicate higher levels of affiliated stigma. The Cronbach alpha for the individual sub-scales in the present study were 0.90 (cognitive stigma), 0.90 (affective stigma), and 0.89 (behavioral stigma). The overall Cronbach alpha in the present study was 0.95.

### Ethical considerations

The study was conducted in accordance with the Declaration of Helsinki 2013. The study received ethical approval from the Research Ethics Committee of the Mbarara University of Science and Technology (#29/03-20). The Psychiatry Department at MRRH granted permission to collect data from the participants. All participants provided voluntary written informed consent to participate in the study.

### Statistics analysis

All data were entered in STATA version 16 for cleaning and formal analysis. For descriptive statistics, means and standard deviations were used to summarize continuous variables, while proportions and percentages were used to summarize categorical variables. The mediating effect of affiliated stigma was tested using the four steps of Baron and Kenny (1986) [27]. In Step 1, the predictor variable (i.e., caregivers’ involvement) must be significantly associated with the outcome variable (i.e., caregivers’ burden). In Step 2, the predictor variable (i.e., caregivers’ involvement) must be significantly associated with the potential mediating variable (i.e., affiliated stigma). Step 3 involves a simultaneous multiple regression, where the outcome variable (i.e., caregivers’ burden) is regressed on both the predictor variable (i.e., caregivers’ involvement) and the potential mediating variable (i.e., affiliated stigma). In addition, two features must also be met: (i) the potential mediator has to be a significant predictor of the outcome variable, and (ii) the regression coefficients between the predictor and the outcome variables have to be reduced in relation to those in Step 1. In Step 4, the regression coefficients in Step 1 and Step 3 have to be significantly different. In the mediation analysis, the socio-demographic characteristics of the caregivers were controlled for.

## Results

### Study participants’ characteristics

A total of 428 informal caregivers participated in the study. The mean age of participants was 39.6 years (SD±14.6), and 62.1% were females. Two-thirds of the participants resided in rural areas (65.6%, *n*=277), and the majority were married (55.5%, *n*=234). Only 15.4% of the caregivers had no formal education (Table 1).

**Table 1:** Description of the socio-demographic characteristics

**Variable**	**n (%) or Mean ± SD**
Age in years	39.6±14.6
*Gender*	
Male	160 (37.9)
Female	262 (62.1)
*Marital status*	
Single	88 (20.8)
Married/Cohabiting	270 (64.0)
Separated	21 (5.0)
Divorced	4 (1.0)
Widowed	39 (9.2)
*Area of residence*	
Urban	145 (34.4)
Rural	277 (65.6)
*Education level*	
No formal education	65 (15.4)
Primary level	160 (37.9)
Secondary level	107 (25.4)
Tertiary	90 (21.3)
*Birth order*	
First born	105 (24.9)
Second born	73 (17.3)
Third born	64 (15.2)
Fourth born	43 (10.2)
Fifth (or below) born	137 (32.5)
Number of children	3.7±3.0
Monthly household income (in USD)	89.9±246.7
Number of patients with mental health illness under their care	1.1±0.4
Period of care for the patient in years	12.1±11.4

### Diagnoses of the patients receiving informal caregiving

The majority of the participants were caring for patient with a mood disorder (45.0%), followed by disorders due to substance use and addictive behaviors (27.2%), and schizophrenia and other primary psychotic disorders (25.8%) (Table 2).

**Table 2:** Psychiatric diagnosis of the patients receiving informal caregiving

**Diagnosis**	**n (%)**
*Neurodevelopmental disorders (6A0)*	
No	414 (98.1)
Yes	8 (1.9)
*Schizophrenia and other primary psychosis (6A2)*	
No	313 (74.2)
Yes	109 (25.8)
*Catatonia (6A4***)**	
No	416 (98.6)
Yes	6 (1.4)
*Mood disorders (6A8)*	
No	232 (55.0)
Yes	190 (45.0)
*Anxiety and fear-related disorders (6B0)*	
No	415 (98.3)
Yes	7 (1.7)
*Disorder due to substance use (6C4) and addictive behaviors (6C5)*	
No	307 (72.8)
Yes	115 (27.2)
*Neurocognitive disorders (6D7)*	
No	407 (96.5)
Yes	15 (3.5)
*Mental and behavioral disorders associated with pregnancy, childbirth, and the puerperium (6E2)*	
No	415 (98.3)
Yes	7 (1.7)

### Affiliated stigma

The mean total score of affiliated stigma was 26.0 out of 88 (SD± 21.8), with a median of 22. The kurtosis (2.7) was below 7, and the skewness (0.7) was below 2, so it can be assumed that affiliated stigma total scores were normally distributed [28]. Among the three components of the affiliated stigma, the mean scores were 1.10 (out of 4) for cognitive stigma (*SD*=1.11), 1.52 (out of 4) for affective stigma (*SD*=1.18), and 0.96 (out of 4) for behavioral stigma (*SD*=0.99).

### Caregiver involvement

The mean score of caregivers’ involvement was 2.87 (out of 5; SD± 0.76). The kurtosis (2.9) was below 7, and the skewness (-0.1) was below 2, so it can be assumed that caregivers’ level of involvement scores were normally distributed. The mean score for four subscales were 2.44 (out of 5) for tension (SD=1.00), 2.90 (out of 5) for supervision (SD=1.07), 2.92 (out of 5) for worrying (SD=1.05), and 3.35 (out of 5) for urging (SD=0.99).

### Caregiver burden

The caregivers’ burden scores ranged between 0 and 88, and only two individuals scored 0 and four individuals scored 88. The mean total score was 38.11(out of 88; SD=21.06). The kurtosis (2.2) was below 7, and the skewness (0.3) was below 2, so it can be assumed that caregivers’ burden scores were normally distributed. A total of 108 had little or no burden (25.6%), 112 had mild to moderate burden (28.9%), 126 had moderate to severe (29.9%), and 66 had severe burden (15.6%).

### Correlation between caregivers’ involvement, burden, and affiliated stigma

All the study variables were significantly highly correlated with each other (correlation coefficient [*r*] ranged from 0.63 [between affiliated stigma and caregivers’ involvement] to 0.71 [between the burden of caregivers and the involvement of caregivers]) (*p*<0.001) (Table 3).

**Table 3:** Correlations between caregivers’ affiliated stigma, involvement, and burden

Variable	**mean ± SD**	**Affiliated stigma**	**Caregivers’ involvement**	**Caregivers’ burden**
Affiliated stigma	26.0 ± 21.8	1		
Caregivers’ involvement	2.87 ± 0.76	0.63*	1	
Caregivers’ burden	38.11 ± 21.06	0.71*	0.71*	1

^*^ *p*<0.001

### Mediating effect testing

#### Step 1

To meet the criteria for mediation, regression analysis between the predictor variable (i.e., caregivers’ involvement) and the outcome variable (i.e., caregivers’ burden) was performed. This showed a significant positive association between the two variables (β=0.03, *p*<0.001), thus meeting the criteria.

#### Step 2

In the second step, the predictor variable (i.e., caregivers’ involvement) was regressed with the potential mediating variable (i.e., affiliated stigma). Again, there was a significant positive association between the two variables following regression (β=15.97, *p*<0.001). Thus, meeting this criterion for this step.

#### Step 3

In the third step, a simultaneous multiple regression was run, where the outcome variable (i.e., caregivers’ burden) was regressed on both the predictor variable (i.e., caregivers’ involvement) and the potential mediating variable (i.e., affiliated stigma) (Additional file 1: Appendix A). Caregivers’ involvement explained 54.6% of the variance in caregivers’ burden, and when affiliated stigma was entered into the regression, it further explained 9.8% of the variance. The potential mediator was a significant predictor of the outcome variable (β=0.03, *p*<0.001), and the regression coefficient between predictor and outcome variable was reduced (*β*=0.02, *p*<0.001). Female gender significantly reduced the burden of caregiving (β= -0.23, *p*<0.001) and no other sociodemographic characteristics was associated with caregivers’ burden. The regressions involved in testing for the mediating roles are presented in Table 4.

**Table 4:** Testing for affiliated stigma as a mediator using the four steps of Baron and Kenny

**Step**	**β**	**95% confidence interval**	***p*-value**
Step 1			
Outcome: caregivers’ burden			
Predictor: caregivers’ involvement	0.03	0.02 – 0.03	<0.001
Step 2			
Outcome: Caregivers’ involvement			
Predictor: Affiliated stigma	15.97	14.09 – 17.85	<0.001
Step 3 a (Multivariate regression while controlling for social demographics)			
Outcome: Caregivers’ burden			
Predictor: Caregivers’ involvement	0.03	0.02 – 0.03	<0.001
Step 3b (Multivariate regression while controlling for social demographics)			
Outcome: Caregivers’ burden			
Mediator: Affiliated stigma	0.40	0.32 – 0.47	<0.001
Predictor: Caregivers’ involvement	0.02	0.01 – 0.02	<0.001

#### Step 4

The fourth step required the regression coefficients in Step 1 and Step 3 to be significantly different. Again, there was a significant difference between the regression coefficients (χ^2^=73.62, *p*<0.001), indicating that affiliated stigma was a mediator between caregivers’ burden and involvement.

## Discussion

The present study demonstrated the association between caregivers’ involvement, affiliated stigma, and the burden of caregiving among caregivers of individuals with mental illness in Uganda. Affiliated stigma was found to fully mediate the relationship between caregivers’ involvement and the burden of caregiving. Among the sociodemographic characteristics, being female was associated with a decreased severity of the burden of caregiving. The findings in the present study are partially consistent with a study among caregivers of mental health patients in China, where affiliated stigma had a mediating role in the caregiver’s burden but with another psychological aspect of face concern (i.e., a salient construct regarding social representations of an individual's desire to preserve and maintain their social image and social worth, based on their specific role within the interpersonal context) [6]. When involved in patient care, the feeling of stigmatization among caregivers begins as a result of the patient’s changed behaviors due to mental illness [11, 29]. Community members' negative attitudes towards their relatives with mental health illnesses can also increase caregivers’ feelings of stigmatization and guilt [29]. Due to the challenges of their roles as caregivers, people who provide care for those who have mental illnesses ultimately experience distress and frustration in regard to their caregiving responsibilities [30, 31]. Moreover, in addition to the burden of caregiving, majority of the primary caregivers lack support, and live under fear of judgment from society [32]. Other research has reported a link between displays of aggressive behavior and an increased caregiver burden and affiliate stigma [33, 34]. Such conditions may increase the caregiving burden, and therefore, the mediating effect of affiliated stigma. Change in a patient's behavior may require additional time and support from their caregivers (i.e., caregiver involvement) to support and prevent the patient from being perceived as a burden to the community or other relatives. Mental illness being a chronic condition requires prolonged involvement of the caregivers to support the patients in adhering to their medicines for continued stability.

However, a study by Mak and Cheung found no relationship between involvement and affiliated stigma of the caregivers (i.e., their involvement does not lead to negative perceptions that may lead to stigma or increased burden of care) [4]. The difference between the present study results and that of Mak and Cheung’s study may be due to the level of involvement required by the caregivers in their respective countries of Uganda and China [4]. Uganda is a low-income country with a poorly developed mental health system that is more highly dependent upon informal caregivers than in China. This results in many more duties for caregivers in Uganda (compared to China), and many may end up feeling the burden of caregiving and affiliated stigma [35, 36].

Contrary to other studies [37, 38], being female in the present study was associated with a decreased severity of the burden of caregiving. Women are often the main informal caregivers in many parts of the world and are often seen as the best caregivers in a situation where care is more intense and requires more problem-solving skills [37]. The contrary findings may be due to the fact that women in Uganda have better-coping skills than men – something attributed to the fact that women provide most of the day to day caregiving roles which stems from their nurturing and upbringing [39, 40] through which they gain skills on how to handle stressful situations and learn problem-solving skills [41]. The reduced burden of caregiving reported by women in this study could also be due to the social desirability bias related to cultural and social expectations of women in most African settings where women are expected to provide care and put everyone first before themselves [42, 43] hence the positive responses in this study. On the other hand, fathers are often absent from home due to work and search for money, and hence not invested in the caregiving roles [41]. However, research has documented that support and involvement of men in the care of a family member with mental illness can improve the individual's mental health outcomes and reduce the risk of relapse [44]. Also, compared to men, many women in Uganda are engaged in activities that may help relieve the burden of caregiving, such as being involved in religious and spiritual groups/communities for prayers and social support [41, 45, 46].

Caregivers of patients with schizophrenia have reported that their spirituality and religious beliefs helped them cope positively with caregiving stress [47]. Other research has shown that mindfulness based cognitive therapy has been used to improve resilience among women caregivers which helped them cope effectively with the caregiving stress and other associated challenges [48]. Overall, mindfulness based approaches have potential to improve important aspects of resilience including problem solving skills, improved self-esteem, coping and social skills [48]. Emotional regulation training interventions have also been documented to reduce stress levels and regulate emotions among caregivers of patients with schizophrenia [49]. More literature points out that support given to the caregivers of patients with mental illness most especially from family members reduces caregiver burden and lowers levels of emotional distress [50]. A study by Su and Chang (2020) indicated that male caregivers experience a higher burden of care and higher levels of affiliated stigma compared to female caregivers [9] while other research has shown that women caregivers experience higher levels of caregiver burden than men [51, 52]. Studies indicating higher caregiver burden and distress among women caregivers argue that women lack support during their caregiving roles [53, 54]. Moreover, it has been documented that caregivers of patients with mental illness have little or no involvement with the health care system which worsens their caregiving burden especially when they don’t know who to turn to when they need advice in their caregiving roles [55]. Mental health services should be extended to both the patients and their caregivers for better treatment outcomes [56].

With affiliated stigma, caregiver involvement, and caregiver burden all being linked in the current study, previous research has suggested strategies that can be used to reduce affiliated stigma among caregivers of patients with mental illnesses and improve caregiver mental wellbeing, thereby improving patient care. For example, mental health literacy interventions that use multicomponent approaches like psychoeducation, equipping caregivers with coping strategies to combat affiliated stigma, and having group therapy sessions with other caregivers can help in alleviating the burden of care [57]. Education about mental health stigma and other techniques like yoga, meditation, and relaxation are additional interventions that can be utilized to enhance caregivers' general wellness and quality of life [57]. Psychoeducation and general education programs aimed at reducing anxiety have also been found to lower caregiver burden and improve social functioning [58]. Mental health professionals should consider providing educational opportunities to the caregivers of people with mental illness to improve their knowledge of mental illness and provide information on appropriate care of patients in the community, and home environment, communication skills, and available resources within the communities [33, 58]. Interventions aimed at providing counselling to caregivers by mental health professionals and establishing family support groups have also been shown to help alleviate stigma among care givers [31].

Several limitations should be noted when considering the study findings. First, the present study was cross-sectional in nature, where determining causality among variables is not possible. Therefore, longitudinal studies are recommended to evaluate caregivers' experiences and provide further insight concerning the relationship between the development of affiliated stigma, the burden of caregiving, and mental health illness. Secondly, the psychometric instruments used have not been validated for use in Uganda specifically. Future research should validate these tools to make them more culturally appropriate. Third, the study relied on self-reported data, which may have introduced various biases into the data (e.g., memory recall and social desirability). Therefore, further studies using different methodologies, such as longitudinal studies involving multiple sites, are needed to confirm the findings here. Lastly, generalizability is limited because all participants were recruited from the Mbarara Regional Referral Hospital (MRRH) Psychiatry Department. Despite these limitations, the present study provides useful benchmark data to initiate further research on the relationship between caregivers’ involvement, burden, and affiliated stigma.

## Conclusion

Affiliated stigma was found to be a full mediator between caregivers’ roles and their involvement. The findings in the present study suggest that interventions to address affiliated stigma should be incorporated into mainstream mental health care to reduce the burden of caregiving. For effective interventions to reduce affiliated stigma, further studies are recommended to develop culturally acceptable methods incorporating strategies that target key variables associated with caregiving (e.g., gender).

## Supplementary Information

**Additional file 1:** **Appendix A.** Multivariable regression model showing the association between caregivers’burden and caregivers’ involvement with affiliated stigma as a mediator 

**Additional file 2.**

## Data Availability

The datasets used and/or analyzed during the current study have been attached as Additional file 2.

## Abbreviations

IEQ: Involvement Evaluation Questionnaire
SD: Standard deviation
MRRH: Mbarara Regional Referral Hospital

## References

[1] Henderson C, Evans-Lacko S, Thornicroft G (2013). Mental illness stigma, help seeking, and public health programs. Am J Public Health.

[2] Subu MA, Wati DF, Netrida N, Priscilla V, Dias JM, Abraham MS, Slewa-Younan S, Al-Yateem N (2021). Types of stigma experienced by patients with mental illness and mental health nurses in Indonesia: a qualitative content analysis. Int J Ment Health Systems.

[3] Stigma, Prejudice and Discrimination Against People with Mental Illness [https://www.psychiatry.org/patients-families/stigma-and-discrimination#:~:text=Public%20stigma%20involves%20the%20negative,have%20about%20their%20own%20condition].

[4] Mak WWS, Cheung RYM (2008). Affiliate stigma among caregivers of people with intellectual disability or mental illness. J Appl Res Intellect Disabil.

[5] Corrigan PW, Watson AC (2002). Understanding the impact of stigma on people with mental illness. World Psychiatry.

[6] Mak WW, Cheung RY (2012). Psychological distress and subjective burden of caregivers of people with mental illness: the role of affiliate stigma and face concern. Community Ment Health J.

[7] Kahn PV, Wishart HA, Randolph JS, Santulli RB (2016). Caregiver stigma and burden in memory disorders: an evaluation of the effects of caregiver type and gender. Curr Gerontol Geriatrics Res.

[8] Werner P, Mittelman MS, Goldstein D, Heinik J (2012). Family stigma and caregiver burden in Alzheimer's disease. Gerontologist.

[9] Su JA, Chang CC. Association between family caregiver burden and affiliate stigma in the families of people with dementia. Int J Environ Res Public Health. 2020;17(8):2772. 10.3390/ijerph17082772 PMC7215659 32316454

[10] Harris GM, Durkin DW, Allen RS, DeCoster J, Burgio LD (2011). Exemplary care as a mediator of the effects of caregiver subjective appraisal and emotional outcomes. Gerontologist.

[11] Hilgeman MM, Allen RS, DeCoster J, Burgio LD (2007). Positive aspects of caregiving as a moderator of treatment outcome over 12 months. Psychol Aging.

[12] González-Torres MA, Oraa R, Arístegui M, Fernández-Rivas A, Guimon J (2007). Stigma and discrimination towards people with schizophrenia and their family members. A qualitative study with focus groups. Soc Psychiatry Psychiatr Epidemiol.

[13] Larson JE, Corrigan P (2008). The stigma of families with mental illness. Acad Psychiatry.

[14] Chang KH, Horrocks S (2006). Lived experiences of family caregivers of mentally ill relatives. J Adv Nurs.

[15] Tsang HW, Tam PK, Chan F, Cheung WM (2003). Sources of burdens on families of individuals with mental illness. Int J Rehab Res.

[16] Dooley WK, Shaffer DR, Lance CE, Williamson GM (2007). Informal care can be better than adequate: development and evaluation of the exemplary care scale. Rehab Psychol.

[17] Karp DA, Tanarugsachock V (2000). Mental illness, caregiving, and emotion management. Qual Health Res.

[18] Schuster F, Holzhüter F, Heres S, Hamann J (2020). Caregiver involvement in psychiatric inpatient treatment - a representative survey among triads of patients, caregivers and hospital psychiatrists. Epidemiol Psychiatric Sci.

[19] Martín J, Padierna A, van Wijngaarden B, Aguirre U, Anton A, Muñoz P, Quintana JM (2015). Caregivers consequences of care among patients with eating disorders, depression or schizophrenia. BMC Psychiatry.

[20] Platt S (1985). Measuring the burden of psychiatric illness on the family: an evaluation of some rating scales. Psychol Med.

[21] Martínez-Mesa J, González-Chica DA, Bastos JL, Bonamigo RR, Duquia RP (2014). Sample size: how many participants do I need in my research?. An Bras Dermatol.

[22] Giacco D, Fiorillo A, Del Vecchio V, Kallert T, Onchev G, Raboch J, Mastrogianni A, Nawka A, Hadrys T, Kjellin L (2012). Caregivers' appraisals of patients' involuntary hospital treatment: European multicentre study. Br J Psychiatry.

[23] Alzahrani SH, Fallata EO, Alabdulwahab MA, Alsafi WA, Bashawri J (2017). Assessment of the burden on caregivers of patients with mental disorders in Jeddah, Saudi Arabia. BMC Psychiatry.

[24] Al-Rawashdeh SY, Lennie TA, Chung ML (2016). Psychometrics of the Zarit burden interview in caregivers of patients with heart Failure. J Cardiovasc Nurs.

[25] Bédard M, Molloy DW, Squire L, Dubois S, Lever JA, O'Donnell M (2001). The Zarit Burden Interview: a new short version and screening version. Gerontologist.

[26] Okewole A, Dada MU, Ogun O, Bello-Mojeed M, Usoh T (2011). Prevalence and correlates of psychiatric morbidity among caregivers of children and adolescents with neuropsychiatric disorders in Nigeria. Afr J Psychiatry (Johannesbg).

[27] Baron RM, Kenny DA (1986). The moderator–mediator variable distinction in social psychological research: conceptual, strategic, and statistical considerations. j pers soc psychol.

[28] Curran PJ, West SG, Finch JF (1996). The robustness of test statistics to nonnormality and specification error in confirmatory factor analysis. Psychol Methods.

[29] Iseselo MK, Kajula L, Yahya-Malima KI (2016). The psychosocial problems of families caring for relatives with mental illnesses and their coping strategies: a qualitative urban based study in Dar es Salaam, Tanzania. BMC Psychiatry.

[30] Hailemariam KW (2015). The psychological distress, subjective burden and affiliate stigma among caregivers of people with mental illness in Amanuel Specialized Mental Hospital. Am J Appl Psychol.

[31] Girma E, Möller-Leimkühler AM, Dehning S, Mueller N, Tesfaye M, Froeschl G (2014). Self-stigma among caregivers of people with mental illness: toward caregivers’ empowerment. J Multidiscip Healthc.

[32] Shi Y, Shao Y, Li H, Wang S, Ying J, Zhang M, Li Y, Xing Z, Sun J (2019). Correlates of affiliate stigma among family caregivers of people with mental illness: a systematic review and meta-analysis. J Psychiatr Ment Health Nurs.

[33] Ebrahim OS, Al-Attar GS, Gabra RH, Osman DM (2020). Stigma and burden of mental illness and their correlates among family caregivers of mentally ill patients. J Egypt Public Health Assoc.

[34] Phillips MR, Pearson V, Li F, Xu M, Yang L (2002). Stigma and expressed emotion: a study of people with schizophrenia and their family members in China. Br J Psychiatry.

[35] Kaggwa MM, Najjuka MS, Kesande C, Nyemara N, Kule M, Mamum MA, Bongomin F, Ashaba S (2022). Length of stay of hospitalized patients at tertiary psychiatry facilities in Uganda: the role of caregiver’s presence. Discover Ment Health.

[36] Kaggwa MM, Harms S, Mamun MA (2022). Mental health care in Uganda. Lancet Psychiatry.

[37] Swinkels J, Tilburg TV, Verbakel E, Broese van Groenou M (2019). Explaining the gender gap in the caregiving burden of partner caregivers. J Gerontol Series B.

[38] Sharma N, Willen E, Garcia A, Sharma TS (2014). Attitudes toward transitioning in youth with perinatally acquired HIV and their family caregivers. J Assoc Nurse AIDS Care.

[39] Kipp W, Tindyebwa D, Rubaale T, Karamagi E, Bajenja E (2007). Family caregivers in rural Uganda: the hidden reality. Health Care Women Int.

[40] Taylor L, Seeley J, Kajura E. Informal care for illness in rural southwest Uganda: the central role that women play. Health Transit Rev. 1996;6(1):49-56. 10163411

[41] Kaggwa MM, Muwanguzi M, Nduhuura E, Kajjimu J, Arinaitwe I, Kule M, Najjuka SM, Rukundo GZ (2021). Suicide among Ugandan university students: evidence from media reports for 2010–2020. BJPsych Int.

[42] Pharr JR, Dodge Francis C, Terry C, Clark MC: Culture, caregiving, and health: exploring the influence of culture on family caregiver experiences. Int Scholarly Res Notices. 2014;2014:1.

[43] Ng R, Indran N (2021). Societal perceptions of caregivers linked to culture across 20 countries: evidence from a 10-billion-word database. PLoS One.

[44] Chen S, Wang Y, Liu Y (2017). The role of men in the caregiving of family members with mental illness: a systematic review. J Ment Health.

[45] Rathier LA, Davis JD, Papandonatos GD, Grover C, Tremont G (2015). Religious coping in caregivers of family members with dementia. J Appl Gerontol.

[46] Triana L, Sudjatmiko IG. The role of religious coping in caregiving stress. Religions. 2021;12(6):440.

[47] Gojer A, Gopalakrishnan R, Kuruvilla A (2018). Coping and spirituality among caregivers of patients with schizophrenia: A descriptive study from South India. Int J Cult Mental Health.

[48] Solati K (2017). The efficacy of mindfulness-based cognitive therapy on resilience among the wives of patients with schizophrenia. J Clin Diagn Res.

[49] Behrouian M, Ramezani T, Dehghan M, Sabahi A, Ebrahimnejad Zarandi B (2020). The effect of emotion regulation training on stress, anxiety, and depression in family caregivers of patients with schizophrenia: a randomized controlled trial. Community Mental Health J.

[50] Gonzalez JM, Perlick DA, Miklowitz DJ, Kaczynski R, Hernandez M, Rosenheck RA, Culver JL, Ostacher MJ, Bowden CL (2007). Factors associated with stigma among caregivers of patients with bipolar disorder in the STEP-BD study. Psychiatric services.

[51] Yee JL, Schulz R (2000). Gender differences in psychiatric morbidity among family caregivers: a review and analysis. The Gerontologist.

[52] Caqueo-Urízar A, Miranda-Castillo C, Giráldez SL, Maturana SIL, Pérez MR, Tapia FM (2014). An updated review on burden on caregivers of schizophrenia patients. Psicothema.

[53] Akpınar B, Küçükgüçlü Ö, Yener G (2011). Effects of gender on burden among caregivers of Alzheimer's patients. J Nursing Scholar.

[54] Caqueo-Urízar A, Gutiérrez-Maldonado J (2006). Burden of care in families of patients with schizophrenia. Qual Life Res.

[55] Ewertzon M, Andershed B, Svensson E, Lützén K (2011). Family member's expectation of the psychiatric healthcare professionals' approach towards them. J Psychiatr Ment Health Nurs.

[56] El-Tantawy AMA, Raya YM, Zaki A (2010). Depressive disorders among caregivers of schizophrenic patients in relation to burden of care and perceived stigma. Curr Psychiatry.

[57] Monnapula-Mazabane P, Babatunde GB, Petersen I (2021). Current strategies in the reduction of stigma among caregivers of patients with mental illness: a scoping review. S Afr J Psychol.

[58] Akbari M, Alavi M, Irajpour A, Maghsoudi J (2018). Challenges of family caregivers of patients with mental disorders in Iran: a narrative review. Iran J Nurs Midwifery Res.

