# Supplementary material for: Involvement and burden of informal caregivers of patients with mental illness: the mediating role of affiliated stigma
Source: BMC Psychiatry. 2023 Jan 26;23:72. doi: 10.1186/s12888-023-04553-x (PMC9881274; doi:10.1186/s12888-023-04553-x)
Supplement: Supplementary file 1 — Additional file 1: Appendix A. Multivariable regression model showing the association between caregivers’burden and caregivers’ involvement with affiliated stigma as a mediator [file 12888_2023_4553_MOESM1_ESM.docx]

**Appendix A: Multivariable regression model showing the association between caregivers’ burden and caregivers’ involvement with affiliated stigma as a mediator**

| **Variable** | **Multiple regression with only predictor variable** | | **Multiple regression with predictor variable and potential mediator** | |
| --- | --- | --- | --- | --- |
|  | **B (*SE*)** | ***p*-value** | **B (*SE*)** | ***p*-value** |
| **Burden of caregivers** | **0.03 (0.01)** | **<0.001** | **0.02 (0.01)** | **<0.001** |
| **Affiliated stigma** |  |  | **0.40 (0.04)** | **<0.001** |
| Age | -0.01 (0.01) | 0.989 | -0.01 (0.01) | 0.985 |
| *Gender* | | | | |
| Male | 1 |  | 1 |  |
| Female | -0.27 (0.07) | <0.001 | -0.23 (0.06) | <0.001 |
| *Level of education* | | | | |
| No formal education | 1 |  | 1 |  |
| Primary level | 0.01 (0.10) | 0.968 | 0.02 (0.09) | 0.802 |
| Secondary level | -0.19 (0.12) | 0.100 | -0.09 (0.10) | 0.377 |
| Tertiary | -0.14 (0.12) | 0.254 | -0.01 (0.11) | 0.889 |
| *Marital status* | | | | |
| Single | 1 |  | 1 |  |
| Married/cohabiting | 0.18 (0.11) | 0.089 | 0.14 (0.09) | 0.135 |
| Separated | 0.23 (0.18) | 0.196 | 0.26 (0.16) | 0.100 |
| Divorced | 0.33 (0.35) | 0.349 | 0.39 (0.31) | 0.203 |
| Widowed | 0.42 (0.10) | 0.017 | 0.27 (0.16) | 0.083 |
| *Birth order* | | | | |
| First born | 1 |  | 1 |  |
| Second born | -0.04 (0.10) | 0.657 | -0.07 (0.09) | 0.459 |
| Third born | -0.17 (0.11) | 0.105 | -0.20 (0.09) | 0.055 |
| Fourth born | -0.05 (0.12) | 0.664 | -0.10 (0.11) | 0.356 |
| Fifth (or below) born | -0.09 (0.09) | 0.291 | -0.08 (0.08) | 0.288 |
| Number of children | -0.02 (0.02) | 0.403 | -0.01 (0.02) | 0.523 |
| Monthly household income | -0.01 (0.01) | 0.543 | 0.01 (0.01) | 0.915 |
| Number of patients with mental health illness under their care | 0.05 (0.08) | 0.531 | 0.06 (0.07) | 0.388 |
| Period of care for the patient in years | 0.01 (0.01) | 0.729 | 0.01 (0.01) | 0.812 |
